# Topic selectivity and adaptivity promote spreading of short messages

**DOI:** 10.1038/s41598-022-19719-y

**Published:** 2022-09-19

**Authors:** Patryk A. Bojarski, Krzysztof Suchecki, Janusz A. Hołyst

**Affiliations:** Center of Excellence for Complex Systems Research, Faculty of Physics, Warsaw University of Technology, Koszykowa 75, 00-662 Warsaw, Poland

**Keywords:** Complex networks, Computational science

## Abstract

Why is the Twitter, with its extremely length-limited messages so popular ? Our work shows that short messages focused on a single topic may have an inherent advantage in spreading through social networks, which may explain the popularity of a service featuring only short messages. We introduce a new explanatory model for information propagation through social networks that includes selectivity of message consumption depending on their content, competition for user’s attention between messages and message content adaptivity through user-introduced changes. Our agent-based simulations indicate that the model displays inherent power-law distribution of number of shares for different messages and that the popular messages are very short. The adaptivity of messages increases the popularity of already popular messages, provided the users are neither too selective nor too accommodating. The distribution of message variants popularity also follows a power-law found in real information cascades. The observed behavior is robust against model parameter changes and differences of network topology.

## Introduction

Information has a profound impact on our life. It can influence opinions and shape collective decisions, including voting^1–3^ or behavior in crisis situations^4^. This is especially true in recent years, with social networks becoming one of if not the most important source of information for people^5^. It is therefore of high importance to understand the process of information spreading, especially since misinformation - false or highly biased information, can significantly impact the outcome of choices a person makes and is potentially open to manipulation^3,6–8^. The topic of information spreading in social networks has been under scrutiny, especially for large online platforms such as Facebook^9^ or Twitter^10,11^. It has been observed that spreading of infectious diseases and information share some similarities—most importantly lack of any sort of conservation laws, unlike diffusion of physical fluids or heat. The simple epidemic models, such as Susceptible-Infected^12^ have been therefore often used to represent and model the spread of information^13^. Similar models, like Independent Cascade models have been also used to model spreading of information^13^, including topic-aware models^14^. It has been shown however, that the real spreading process, depending on the type of the information, could be substantially different from a simple compartmental epidemic model^15^. In fact, many features of the real information spreading processes have been identified^16^ and modeled^17–20^, including applications^21^. A significant research has been made into the impact of message features on the spreading process—in particular, the communication on the Twitter platform^11,22,23^.

It has been shown that people do not consume and spread news uniformly as they receive it, but selectively according to their perception^24^ or their content and interests^11^. The choice of what to read and trust will depend on user’s beliefs and their perception of the message. The selectivity of message consumption will further impact, perhaps to even greater degree, what is actually shared by the user. The existence of such confirmation bias is well known^25^ and it has also been directly shown that it causes individuals not only to choose information sources that confirm what they know, but also selectively peruse stories and messages from the sources they already subscribe to^26,27^. Both selective news consumption and the choice of information sources may lead to creation of so-called echo chambers^27–31^, where like-minded individuals form tight-knit communities that may consume almost no information from outside of their bubble, either by choice or by ignorance. In recent times, due to the existence of automated recommendation systems, the selectivity of information consumption may not be completely a result of individual choice or preference^32–36^.

Aside from choosing what they read and share, users in online network can choose how and exactly what details of the information they obtained they want to share. Not being held by standards of the traditional media^5^, the users can introduce changes or individual biases as they propagate information further to their own friends. This can affect both form and content of messages. The changes will be shaped by user’s beliefs, perceptions and potentially agenda, so the adapted messages will reflect user’s beliefs. Several cases of mutable message spreading have been studied, such as changes in chain-letters^37^, stories propagated through blogs^38^ or online social networking services such as Facebook^39^. It has also been shown experimentally that the meaning of messages can change even if individuals are only trying to change its form, such as message shortening^40^. This phenomenon poses problems in tracking message content spread in social networks, although immutable markers such as URL links^41^ or hashtags^39^ have been used. There was research on tracking changing information, such as specific chain letters^42^ or meme-like phrases by phrase inclusion in another^38^. It has been also shown that spread of changing information shows characteristics similar to genetic mutations^37,43^. In fact, meme variant popularity shows characteristics of the Yule model designed to represent evolving populations^39^.

Unlike a typical epidemic or Independent Cascade model, messages in online networks do not spread independently from each other, which is often dubbed complex contagion^44^. In case of online social media in particular, the users can devote only a limited time to their online activities which results in limited capacity to read and share. Given the immense amount of information present on the internet, this means that effectively different messages compete for the attention of the user and not all messages a user is exposed to will be even read. This issue has been modeled and investigated before^45,46^. It is known in particular that competing epidemics may result in power-law distribution of the cascade sizes^45^. It is worth to note, that such competition may be viewed as a higher-order interactions between users. This approach is subject to extensive research in recent years^47,48^, including with explicit competition between messages^49,50^.

In this work, we propose a new model of information spreading in online social networks, that reflects three aspects of a real process: *selective* consumption and sharing of messages, possibility to *adapt* messages by users spreading them and *competition* for user’s attention between messages. Our primary assumption is that online users want to propagate information they like or agree with, such as a conspiracy theory believer propagating information supporting the conspiracy he believes in, a scientist propagating only scientifically plausible content, or a politician tweeting only information supporting her agenda. If confronted with a message that user does not completely agree with but thinks it still has some merit, he may decide to adjust the information, removing parts of the message that he does not agree with, modify it, or even add something from himself. Such a modification may be motivated by a need to shorten it, share their own thought on the matter, push their own agenda or may be unintentional, resulting from individual understanding based on user’s perceptions and beliefs. The key point here is that different users have different opinions or preferences. Users will forward information similar to their beliefs, while ignoring and effectively stifling information that they do not agree with. Such spreading process may therefore bear some resemblance to opinion dynamics, as messages received and read may in general influence users’ opinions^51^. Here, we opt to look at a short-term process, where we assume opinions and beliefs are constant. They are chosen at the beginning and do not change afterwards. The spreading of messages relies on these opinions, but as the opinions are fixed, the model should not be considered opinion spreading model. We do not consider the veracity of opinions or messages and represent both opinions and message contents by abstract vectors in multidimensional space. This is partially inspired by often used representation of documents as vectors in word space, with cosine between such vectors a measure of similarity^52^. The representation is also similar to previous topic-aware spreading models based on Independent Cascades by Barbieri et al.^14^, but with significant difference as to what the vectors represent. Existing topic-aware model represents a share of a topic in a message as vector components, and is based on real text analysis, while in our case the components represent opinions on abstract topics.

The main aim of the work is to investigate the behavior of the proposed agent-based model and understand the impact of its three features not present in typical epidemic-inspired models: user *selectivity*, message *adaptivity* and *competition* for user attention. Each agent holds a randomly chosen vector of opinions, each component with value 1, $-1$ or 0 representing two opposite opinions on a given topic and neutral stance respectively. These are chosen initially and remain constant throughout a simulation. Agent can either post a new message that perfectly reflects a few of his opinions, represented by a vector with only a few components, or read messages he was exposed to by his neighbors. Agent will read and share the newest of the messages that he finds agreeable, meaning cosine similarity was above the threshold value. When sharing a message, the agent may adapt it, changing one of its components to match agent’s own opinion vector. A more detailed description of the model and its parameters can be found in section “Model”. The proposed model does not attempt to capture the exact complex features of a real information spreading process in detail, and is thus not intended as a predictive model. It is instead an explanatory model containing some chosen characteristics of the real process, but with mechanisms and rules clearly defined and not reliant on data. It is therefore more similar to epidemic or threshold models often used to represent information spreading, than a data-driven model fitted to particular data and evaluated purely on the accuracy of their prediction.

We have performed numerical agent-based simulations of the model for various network topologies, including Erdös-Rényi random graph^53^, Barabási-Albert scale-free network^54^ as well as real social networks (see section “Datasets” for details on real networks used). Our two main measures obtained from numerical simulations are popularity of a message and message length. Message popularity is simply the number of times it has been shared in the social network. This includes all variants of the same message, if it has been modified during the spreading process. Message length is the number of opinions contained in them - number of opinion vector components present in the message. It is not a true length like word count or character count used in Tweets, but we expect that the amount of opinions or topics the message touches will be correlated with the actual length of the message.

Our most interesting observation is the increased popularity of the messages that are short, at least when selectivity is not too low or high. This is an effect of selective message reading and sharing, coupled with competition for attention. Competition has a profound effect, enforcing a power-law distribution of message popularity for a wide range of parameter values. Adaptivity of messages has a smaller impact, but for medium values of selectivity it allows popular, short, viral messages to achieve even greater popularity. These findings are further elaborated in the following section “Results”.

The main contribution of this work is proposing a new model of information spread in social networks, that demonstrates the popularity of short messages in online media and thus indirectly explains popularity of Twitter platform that features short messages by design.

## Results

**Figure 1:**
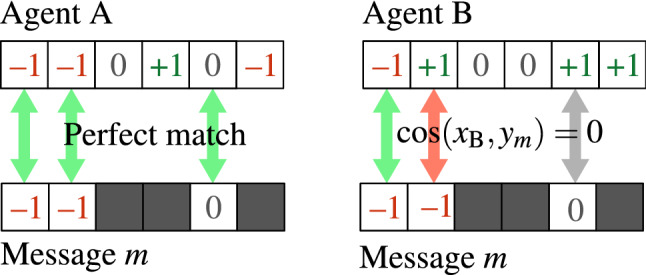
Opinion vectors for agents A and B differ, so message *m* that matches A perfectly will most likely not fit B. An example for opinion vectors with $D=6$ components, with message *m* having length 3.

Our focus was on effects of *selectivity* of users, *adaptivity* of messages and *competition* between them and we investigated these processes using numerical agent-based simulations on several synthetic and real network topologies. Attributes representing opinions of agent *i* are vectors of length *D*, each component $x_{ij}$ having value $-1$,0 or $+1$ representing neutral and two opposing views on a specific topic *j*. In a similar fashion each message *m* is a vector in sub-space of topics, each component $y_{mj}$ also featuring value $-1$, 0 or $+1$ representing message’s content regarding topic *j*. Messages contain only a few out of *D* total possible components, their number being the message length. Figure 1 shows agent’s opinion vectors and message contents in an example. Section “Model” contains a detailed description of the model and its parameters.

Our parameter representing the strength of selectivity is a threshold $\tau \in [-\,1,1]$. If the cosine similarity between agent’s opinion vector and opinion vector of the message is larger than this threshold, the message will be read and shared by the agent, otherwise it will be ignored.

Agents look at messages in order of decreasing freshness until they find one similar enough to share. This means that newer messages may block reading older messages, even if these older messages are closer to agent’s own opinions. In effect, the messages compete for attention, as popular messages will be generally shared more often and thus will have higher chance to be the newest message for agents exposed to them.

The adaptivity is represented through a probability $\alpha$ that when seeing an interesting (with similarity above threshold $\tau$) message, the agent will adapt the message before sharing it. When adapting the message, a single component in the message that is different than corresponding agent’s opinion will be removed or changed to match. Alternatively a single new component reflecting agent’s opinions may be added to the message vector. The adaptivity concerns only message contents and does not affect agent own opinions or network topology.

We have tracked the popularity of messages and their average length (see section “Measures” for details). In particular, the distribution of message popularity has been investigated – the fraction of messages that attained a given total number of shares. The simulations have used synthetic connection topologies of Erdős–Rényi graphs (ER)^53^ (any pair of nodes connected with fixed probability $p=\langle k \rangle / N$), Barabási–Albert networks (BA)^54^ (evolving scale-free network using preferential attachment, with $P(k) \sim k^{-3}$) as well as Facebook and Twitch user networks (see section “Datasets” for details). Opinion vectors for agents were chosen independently and at random, with each component value being $-1$, 0 or 1 with equal probability.

The simulations focus on networks of size $N=600$ nodes and mean degree $\langle k \rangle =6$ for synthetic networks. The probability of agents creating a new message instead of reading and sharing is $\eta$ and we assumed value $\eta =0.1$ for all simulations. The simulations have been performed for two different adaptivity probabilities $\alpha = {0.0}$ (messages cannot be modified) and $\alpha = {0.2}$ (agents can modify messages) and three selectivity $\tau$ values: $-\,0.4$, 0.2 and 0.8. For each parameter set we made 10 independent realizations and aggregated the results into popularity distributions and mean message length depending on popularity.

**Figure 2:**
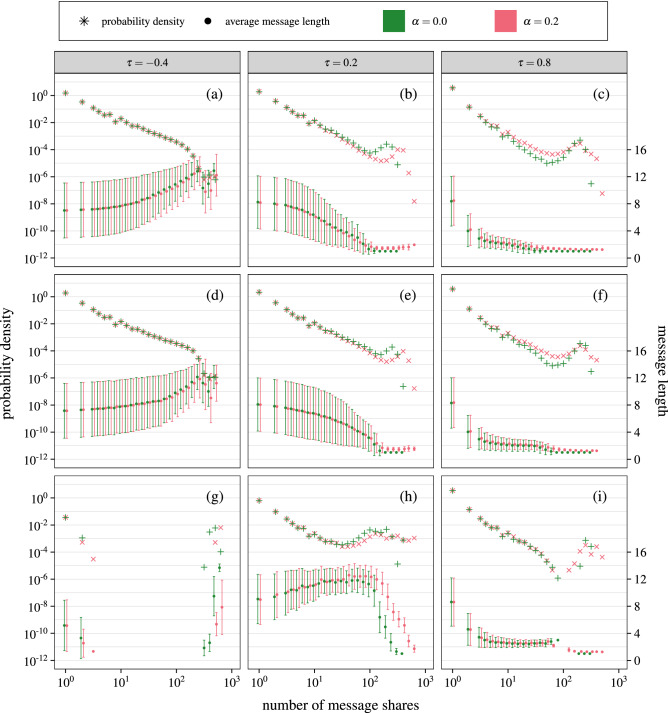
In majority of situations the popularity distribution of messages is a power-law distribution, with the tail dominated by very short messages. The figure shows the influence of user selectivity $\tau$ and message adaptivity $\alpha$ on the popularity and on the average length of messages propagating in synthetic networks. Panels (**a**)–(**f**) show spreading with competition between messages (agents can share only the most recent message he likes), while panels g-i show spreading without this constraint. Panels (**a**)–(**c**) present the popularity for ER networks and ${500,000}$ time steps, d-f for BA networks with ${500,000}$ time steps and g-i for ER network, but with a shorter time horizon—50,000 time steps. The simulations were carried out for networks with size $N = 600$ and an average node degree $\langle k \rangle = 6$. The agents’ opinion vectors were randomly selected. The probability of creating a new message equals $\eta = 0.1$. The probability density of the number of shared messages (left vertical axis) is marked with crosses and pluses. The average length of the message (right vertical axis) with a given popularity is marked with dots, and its standard deviation with error bars. Columns correspond to $\tau =-\,0.4$, 0.2 and 0.8, green symbols are for simulations without adaptivity ($\alpha =0$), while red symbols with adaptive messages ($\alpha =0.2$).

Figure 2 shows the results of numerical simulations for ER and BA synthetic networks and also shows the impact of competition between messages. Note that error bars for average message length represent standard deviation of message lengths of given popularity, not the uncertainty of the mean value, that would be significantly smaller.

The first observation is that in majority of situations, the distribution of number of shares of messages is a power-law distribution. However, when the competition is absent and messages spread independently, then for low similarity (Fig. 2g) messages either disappear right away or spread to entire network, like in an independent cascade model^13^ with spreading probability above percolation threshold^55^. If selectivity is high (Fig. 2i), it limits the popularity of messages, resulting in part of distribution resembling power-law, but only vaguely. We can conclude that the competition between messages is the factor producing the observer power-law distribution of popularity.

The second observation is that for medium ($\tau =0.2$) and high selectivity ($\tau =0.8$) as shown on Fig. 2b, c, e, f, h, i the more popular messages are shorter, with most popular being of length around 1. The opposite is true for low selectivity $\tau =-\,0.4$ (Fig. 2a, d, g). This can be explained. As opinion vectors are random, their cosine similarity with essentially random message vectors tend toward 0 as messages become longer. Thus for $\tau <0$ long messages almost never fail to be accepted, while for $\tau >0$ they almost always are below the threshold.

**Figure 3:**
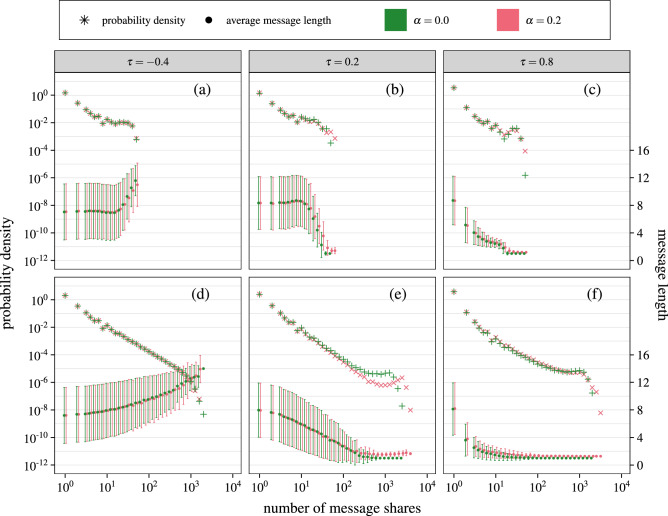
The model behaves in a similar fashion for networks of different sizes. The figure presents the influence of user selectivity $\tau$ and message adaptivity $\alpha$ on the popularity and average length of messages propagating in synthetic BA networks. Panels (**a**)–(**c**) shows message spreading for small networks $N=60$ with ${50,000}$ time steps. Panels (**d**)–(**f**) are for a large BA networks $N=6000$ with ${5,000,000}$ time steps. Other parameters are the same as in Fig. 2. Columns correspond to different selectivity $\tau =-\,0.4$, 0.2, 0.8, green symbols are for simulations without adaptivity ($\alpha =0$) while red are with adaptivity ($\alpha =0.2$).

The third observation is that for medium and high value of selectivity $\tau$ (Fig. 2b, c, e, f, i) we can observe a peak in the popularity distribution, corresponding to viral, short messages. This peak shifts to higher values when adaptivity is introduced and selectivity is medium ($\tau =0.2$, Fig. 2b,e), meaning that already popular messages reach even a larger part of the network. If selectivity is too small, adaptations don’t increase reach, because the messages would spread everywhere anyway if not for competition, and if the selectivity is too high, then any message that is not rejected is already perfect match for the agent and thus can’t be modified.

The fourth observation is that the results for ER random graph (Fig. 2a–c) and BA network (Fig. 2d–f) are very similar. This means that the degree distribution has no noticeable impact on behavior of our model.

To understand whether any of the observed phenomena results from the network size, we have performed numerical simulations for a smaller network of $N=60$ nodes as well as larger network of $N=6000$ nodes, both using Barabási-Albert model topology. The results are shown in Fig. 3.

**Figure 4:**
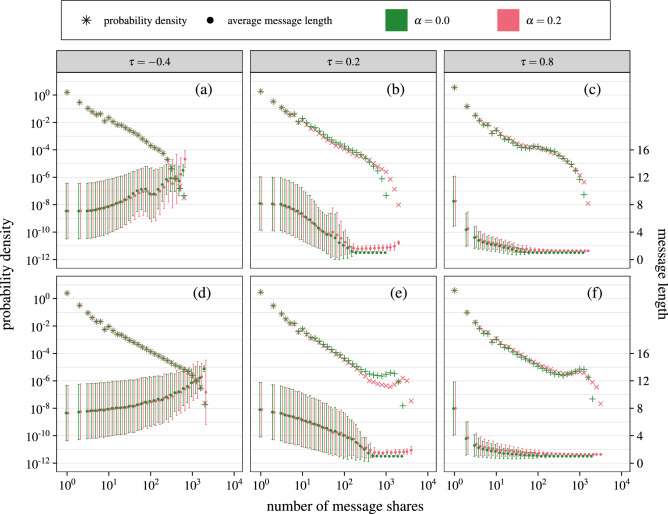
The model behaves similarly in real networks as it does in synthetic networks, although there are visible differences for Facebook ego-network. The figure presents the influence of user selectivity $\tau$ and message adaptivity $\alpha$ on the popularity and average length of messages propagating in real networks. Panels (**a**)–(**c**) shows message spreading for Facebook users network, $N=4039$ and $\langle k \rangle = {43.69}$. while panels (**d**)–(**f**) for English Twich users $N=7126$, $\langle k \rangle = {9.91}$ (see section “Datasets” for dataset details). Number of time steps is equal to ${1,000,000}$ in every case. Columns correspond to different selectivity $\tau =-\,0.4$, 0.2, 0.8, green symbols are for simulations without adaptivity ($\alpha = 0$) while red are with adaptivity ($\alpha = 0.2$).

Comparing these results with Fig. 2d, f it is evident that the behavior of the model is at least qualitatively the same for networks of different sizes. The power-law popularity distribution, the dominance of short messages and adaptivity boosting popularity of already viral messages are all visible. The only different detail for small network is the fact that the peak corresponding to viral messages may not appear (compare Fig. 2e, f with Fig. 3b, c). In consequence the adaptivity impact observed in larger systems may be not visible.

To see whether the topological properties of real social networks alters the behavior of the model, we have included two real network connection topologies in our simulations. We have used an aggregated friendship ego-network of Facebook users and network of friendship of Twitch users, see section “Datasets” for dataset details. In both cases we have only used the network structure and discarded any other user features included in datasets. Agent opinions were assigned randomly, just like for synthetic networks. The results of model simulations on these networks are shown in Fig. 4.

The behavior of the model in the Twitch network (Fig. 4d–f) is very similar to its behavior in a BA network with $N=6000$, which is of a similar size. There is a basic power-law popularity distribution, popular messages are always short and there is a popularity peak corresponding to the viral messages. For Facebook (Fig. 4a–c) however the message length dependence appears to not be as smooth and there is no viral message peak in popularity distribution. This differences may be attributed to a specific, highly modular structure of the Facebook network, that is an aggregation of several ego networks with minimal overlaps (see section“ Datasets” for more details). If messages go viral in some clusters without transferring to another, there will be no sharp popularity peak. This results show that while the behavior is in general robust against network topology, highly modular network structure may alter it.

**Figure 5:**
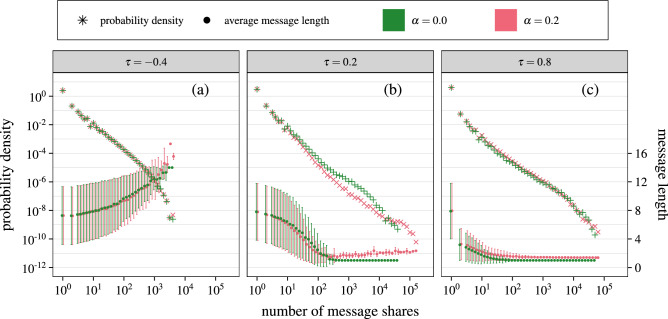
When messages can be re-shared multiple times by the same user, the behavior of the model changes only quantitatively. The graphs presents popularity distribution and average message length for simulations on BA networks of size $N=600$ and for ${500,000}$ time steps. Other parameters are the same as for results shown on Fig. 2. Columns correspond to different selectivity $\tau =-\,0.4$, 0.2, 0.8, green symbols are for simulations without adaptivity ($\alpha =0$) while red are with adaptivity ($\alpha =0.2$).

In all our simulations we have assumed that a specific message may be shared by a given user only once, and will be afterwards ignored, including its variants. This may not always be the case. Users may re-post the same information either unintentionally, because they didn’t recognize it, or intentionally, in hopes of forcefully pushing their views on others. The repeated communications and reinforcement are thought to be behind influence exerted by some of the online content on opinions of users^44^. We have verified the behavior of the model in case messages are allowed to be re-shared any number of times, with results shown at Fig. 5.

The results show that the behavior of the model remains qualitatively the same. The main difference is much broader range of message popularity, since it is not limited to number of users anymore. The viral messages also display a much broader range of popularity and the peak effectively becomes a bump overlaid on the power-law instead of true peak.

**Figure 6:**
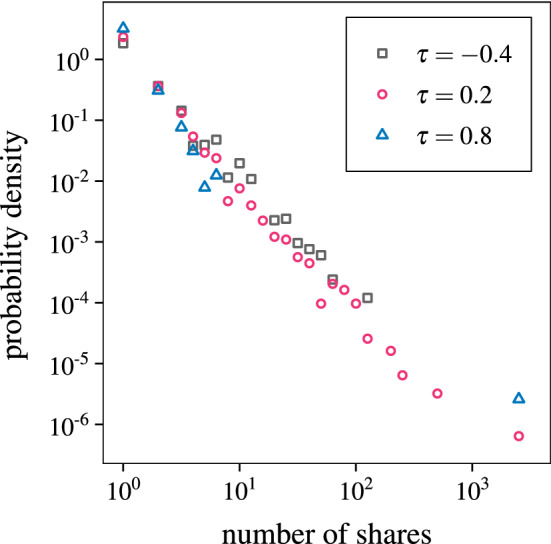
The variants of a single, popular message show a similar power-law distribution of their individual variant popularity. The figure shows the variant popularity distributions for different selectivity parameters $\tau =-0.4$ (gray squares), $\tau =0.2$ (red circles) and $\tau =0.8$ (blue triangles). Simulations were conducted on ER networks with $N = {10,000}$, $\langle k \rangle = 6$ and ${500,000}$ time steps.

Along with the distributions of message popularity in different networks, we have also investigated the distribution of relative popularity of message variants of a single message. Similarly to the work of Adamic et al.^39^, we chose only a single realization, where a single message with the most variants will be analyzed. As seen in Fig. 6, the variant popularity distribution appears to show characteristics of a power-law, meaning that there usually is just one or two main dominant variants, along with a plethora of niche adaptations. This is the same behavior as found in real cascades on Facebook, investigated by Adamic et al.^39^. This shows that our model displays similar characteristics as information cascades found in real social networks.

## Discussion

*Summary*. We have proposed a new, more realistic model of information spreading and shown that selectivity of users and adaptivity of message content promote messages that are short. While the model is simple and abstract by design, it offers a potential explanation as to why short messages are popular. Not only are they easier to read, their content is less often in contradiction to user’s beliefs, allowing the messages to spread more widely than more elaborate, longer messages. This observation may explain popularity of Twitter and similar services that focus users on short messages by design. We also find that the message adaptivity, the ability to change, can increase the popularity of viral messages but only if users are moderately selective about what they share. We have tested the model on synthetic networks, including scale-free Barabási-Albert (BA) network and two real social network topologies. The main conclusion holds regardless of the network size or specific topology features, including degree distribution.

*Topology*. The fact that scale-free degree distribution does not affect the spreading behavior is somewhat unexpected. We anticipated that the existence of hubs in BA networks, coupled with the possibility of changing the content of messages, would allow information to spread more broadly. This is not what we observe. It may be that the randomness of agents’ opinion vectors is responsible. In real social network people tend to keep in touch with like-minded people. Perhaps given different, correlated opinions the hubs would play a more significant role.

*Message length*. As already mentioned, the preference for short messages for positive selectivity may be a potential explanation for the Twitter phenomenon. Artificial limits on message length may have contributed to its popularity as it may have become preferred over longer internet forum or blog posts because it facilitated broader information spread. The research of factors contributing to tweet spreading may not seem to support this claim, showing small positive influence^22^ or statistically insignificant negative^23^ of length on popularity. It must be noted however, that what we mean by “short” messages here is number of topics and related opinions expressed in the message. With Tweets being very short, many of them may express just a single opinion and it may be not directly related to message length counted in characters. In addition, while our model features uncorrelated, static opinions of agents, interests of interacting users, such as followers on Twitter, do change in time to be more uniform^56^. This difference may alter the overall behavior, especially for the small cascades that are most frequent in real systems and may change the perceived influence of factors such as length.

*Variant popularity*. The characteristics of message cascades found in our model are in agreement with observations made in real data. In particular, according to Adamic et al.^39^, the memes on Facebook behave according to a Yule model that describes evolving populations. The popularity distribution of variants of a message (Fig. 6) is qualitatively the same as distributions found by Adamic et al.^39^, regardless of the assumed user selectivity. Our finding that the most popular messages are short is in agreement with another conclusion of Adamic et al.^39^, where the maximum of the popularity occurs for mean lengths significantly below the average (unlike in Twitter research^22,23^ mentioned above). While this is not direct comparison, it still shows that the model shows behavior similar to real social network communication.

*Result comparison*. It is worth to compare the behavior of the model with other, existing models of information spreading. Our model is similar to simple contagion models like Susceptible-Infected (SI) or Independent Cascades model^13^, in that it does not require repeated reinforcement or multiple neighbors to convince user to spread the message, like in the case of complex contagion^44^, for example a threshold model^57^. In fact, in absence of competition, or at very high selectivity, when competition does not play a significant role, our model is very similar to independent cascade model and will produce similar results. The only difference is that independent cascades essentially work like the bond percolation on the network, while in our case, because the similarity is checked for users, it works like the site percolation^58^. When competition is important, the model produces a power-law distribution of popularity for wide range of topologies and model parameters. This is similar to simple competing epidemic processes, where the cascade size distribution also resembles power-law distribution^45^. The behavior of our model is different than the threshold model, often used for spreading of innovations or fads. While threshold model can produce distribution of cascade sizes (popularity) that is similar to a power-law distribution^57^, it does so only for scale-free networks. In our model, the distribution is emergent and does not rely on externally imposed power-law relations.

*Future work*. Our work lays a foundation for possible further research. The first avenue is to make the spreading model richer, featuring correlated user opinions, asymmetric opinion distributions, or the inclusion of heterogeneity in agents’ selectivity or adaptivity. The second avenue is exploiting the obtained results, for example by including a message recommendation system to the model and testing whether its influence could be detected from message spread statistics, similar to how it was possible to do in internet forum discussions^59^. If so, it may be possible to develop a method to detect the presence and assess the influence of recommendation systems in real online social networking services. The third potential research avenue is to implement non-pairwise interactions, presence of which has been discovered in real social networks^47–50,60^.

## Methods

### Model

We assume every agent (user) *i* is connected to $k_i$ other agents, creating a network consisting of *N* users, that represents contacts in the online service along which messages can spread. Each agent has its own opinions on *D* different, independent topics, represented as a vector $\vec{x}$ of length *D*, which we call an opinion vector. Each element *j* has a value $x_{ij} \in \{-1, 0, 1\}$ and corresponds to the same topic for all agents. For example, the third element of any agent’s vector opinion may represent opinion about global warming—whether they believe and care about it ($x_{i3}=1$), deny it ($x_{i3}=-1$) or simply don’t care ($x_{i3}=0$).

We use the asynchronous dynamical rule, picking agent that will act at random, where one unit of time corresponds to *N* single agent updates. When chosen for update, an agent will create a new message with probability $\eta$ and will attempt to read and share messages he is exposed to otherwise.

**Figure 7:**
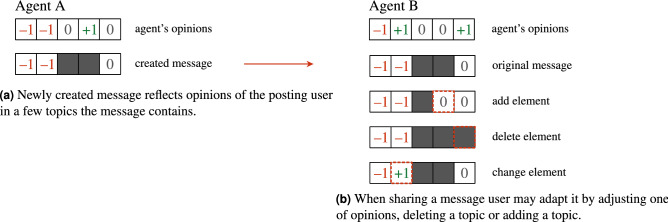
Creation of new messages and possible adaptation of messages when sharing them.

When creating a new message, the agent will immediately share this message with all closest neighbors. Each new message is assigned a unique ID that does not change during the dynamics, even if the message is later changed during the spreading process. We assume that a given message will concern a certain issue (an event, politician’s speech, scientific discovery, etc.) and while its later variants may contain different opinions or paint facts in a different manner, the message will be essentially about the same issue and therefore recognizable. The content of every new message reflects a part of the author’s opinion. We pick message length $d \in [1, {0.15}D]$ at random from uniform distribution and randomly pick *d* topics with equal probabilities to be chosen. The values of opinions of the agent on the selected topics are copied from agent’s opinion vector, forming message content. (Fig. 7a). Thus, the message *y* contains values $y_j \in \{-1,0,1\}$ for a few of the topics and contains no mention about other topics at all.

With a probability $1-\eta$, instead of creating a new message, the user reads the messages he is exposed to, that have been shared by his closest neighbors. It is done in order from the newest to the oldest received. Users do not know when original information was created and only see the time when a message was shared with them. For each message a cosine similarity between the agent’s opinion vector $x_i$ and the message *y* is calculated. Only the components of the opinion vector that are also present in the message itself are taken into account, which is why 0 in a message is not the same as the absence of a topic. If the similarity exceeds a certain threshold $\tau$ (the selectivity parameter), the agent decides he likes it and will share this message with his own neighbors. If, on the other hand, the similarity is equal or less than $\tau$, the user reads increasingly older messages he received, until he finds the first attractive message. If after looking through the whole set of messages received there are none to its liking, the agent simply does not share anything. This means that an agent can only create or share (possibly modifying) one message, to represent a limited capacity to consume and process information. When the euclidean length of either an agent opinion vector or a message is equal to zero, then we assume that the cosine similarity is equal to zero. Note that we obtain a simple SI model when agents share information without considering their opinion (or when we set $\tau = -1$), and they are allowed to share as many messages as they want in one time step.

**Figure 8:**
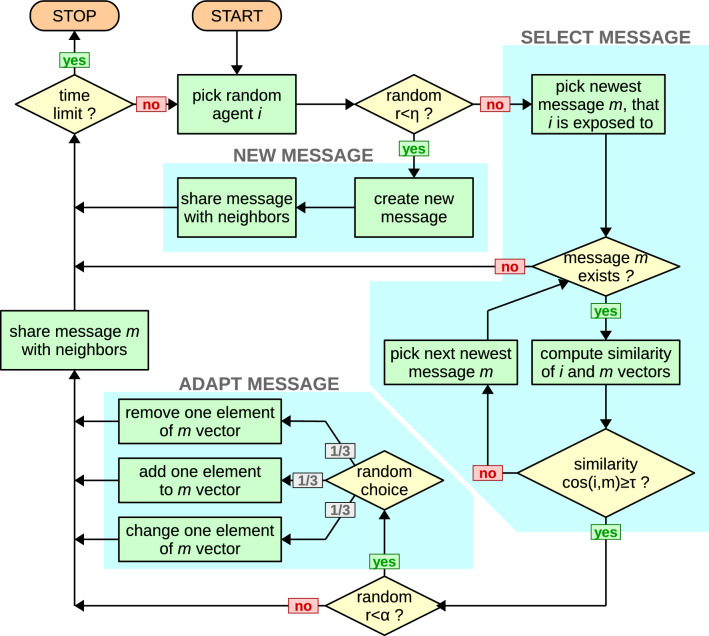
Flowchart of the algorithm for creation, sharing and modification of messages in our model.

Before actually sharing a message that the agent likes, the agent can modify it and create a new message variant. With probability $\alpha$ the message will be adapted in one of three possible ways: (a) adding a new topic *j* to the message, chosen at random and reflecting agent’s opinion $x_{ij}$ on that topic, (b) removing one of topics present in the message that do not reflect his opinions, (c) replacing an opinion on one topic present in the message with his own (as shown on Fig. 7b). Each of the modifications is equally likely, with probability 1/3 to occur, but only a single modification will be done. Users never share the same message (with the same ID) more than once, even if the message has been modified, since it is assumed that it will relate to the same event, even if it paints it in different colors. Users completely ignore any messages with an ID they have already read and considered before.

We finish the dynamics after a pre-determined number of time steps (each consisting of *N* single agent updates). The dynamics can be represented by the diagram as in Fig. 8.

When testing properties of the model we have introduced two alternative models: a model without competition and a model without agent memory.

A model without competition differs from the basic one in that in a single update the agent reads all messages he is exposed to and shares all messages with similarity above $\tau$. This means that each message is always considered, effectively making them spread independently from each other, without competition.

The model without agent memory relaxes the restriction on reading and sharing messages the agent has already shared in the past. Message ID is not remembered, and thus an agent may read and share the same message as many times as it will turn out to be newest message he likes.

### Measures

In our investigation we use the distribution of message popularity and average message length measures. The *popularity distribution* is the distribution of number of times $S_m$ a message *m* has been shared throughout the whole simulation, including all *variants* of the message. The distribution shows the probability density of the number of shares being a certain value *S*. In Fig. 6 (and only there) we use variant popularity instead of message popularity. This measure counts the number of times a specific variant of a message *m* has been shared throughout the simulation, instead of counting all variants of different messages.

*Average length of message* is an unweighted average of the individual message lengths over all messages within a given popularity bin. $\langle L \rangle (S) = \sum _{m : S_m=S} L_m / N_S$, where *S* is number of shares (argument), *m* are different messages, $L_m$ is length of individual message, $S_m$ is number of shares for message *m* and $N_S$ is total number of messages that have number of shares *S*. Individual message length is an average number of topics over all its variants, weighted by number of times given variant was shared. $L_m=\sum _v l_v s_v / \sum _v s_v$ where *v* are variants of message *m*, $l_v$ is length of particular variant and $s_v$ is number of shares of given variant. The error bars on Fig. 2 represent the standard deviation of the distribution of message lengths $L_m$, and does not include variance of lengths between variants of each message, so it does not depend on distribution of $l_v$ for any given message (other than through $L_m$ itself).

### Datasets

We have used two real network topologies during our research: Facebook and Twitch.

*Facebook* dataset we have used is available at Stanford Large Network Dataset Collection^61^ and comes from a paper by McAuley and Leskovec^62^. It is an aggregation of ego-networks of several users, with links being friendship relations between users on Facebook. There is some overlap between individual ego-networks, with almost all nodes belonging to one connected component. The dataset contains $N=4039$ nodes, with $E={88,234}$ edges giving mean degree $\langle k \rangle ={43.69}$. The whole network is highly modular, with modularity^63^ $Q \approx 0.834$ for communities detected using greedy hierarchical algorithm by Blondel et al.^64^. The dataset contains additional data on users, but they have been discarded for our study and only topology of connections has been used.

*Twitch* dataset we have used is also available at Stanford Large Network Dataset Collection^61^ and comes form a paper by Rozemberczki et al.^65^. It contains users of Twitch as nodes and friendship relations between them as links. The dataset contains networks in several languages, but only data for english Twitch is presented in this paper, with $N=7126$ nodes, $E={35,324}$ edges and $\langle k \rangle =9.91$. The dataset contains additional data aside from the network, but it has been discarded for this study and only topology of connections has been used.

## Data Availability

The real network topology data used in our analysis is publicly available at Stanford Large Network Dataset Collection, https://snap.stanford.edu/data. See section “Datasets” for details.

## Code availability

The code for this project is available on GitHub at: https://github.com/PatBoj/mutated-information-spread.
